# An In-Depth Insight into the Profile, Mechanisms, Functions, and Transfer of Essential Amino Acids from Mulberry Leaves to Silkworm *Bombyx mori* L. Pupae and Fish

**DOI:** 10.3390/insects15050332

**Published:** 2024-05-05

**Authors:** Mihaela Hăbeanu, Anca Gheorghe, Georgeta Dinita, Teodor Mihalcea

**Affiliations:** 1 Research Station for Sericulture Baneasa, 013685 Bucharest, Romania; anca.gheorghe@scsbaneasa.ro (A.G.); teodor.mihalcea@scsbaneasa.ro (T.M.); 2 Faculty of Animal Productions Engineering and Management, University of Agronomic Sciences and Veterinary Medicine of Bucharest, 59 Marasti Blvd., District 1, 011464 Bucharest, Romania; georgeta.dinita@usamv.ro

**Keywords:** amino acids, fish, mulberry leaves, proteins, pupae, silkworm

## Abstract

**Simple Summary:**

The silkworm has fascinated people all around the world for centuries due to numerous applications of by-products resulting from its metamorphosis. A decline has been noticed due to industrialization and some disease concerns; however, several reasons strengthen compelling arguments for keeping up this species. The most prevalent nutrients identified along silkworm rearing, from mulberry leaves up to larvae, pupae, and silk, are proteins. The biological value of protein is based on amino acids. The presence of other nutrients, especially fatty acids (n-3 family), vitamins, minerals, and polyphenols, along with the profile in amino acids, which are taken from mulberry leaves and metabolized by larvae and then transferred to pupae, strengthen the conviction according to which silkworm by-products have a great nutritional potential that is either comparable with fish meal or better than other aquaculture-specific feedstuffs. This paper aims to present deep knowledge about amino acids (classification, metabolism, occurrence, mechanisms, and potential functions) for silkworms and fish to further understand these species.

**Abstract:**

The silkworm *Bombyx mori*, the second most varied group of insects, is a fascinating insect that belongs to the Lepidoptera species. We aimed to deepen our knowledge about the composition and significance of amino acids (AA) from the sericulture chain to fish. AAs are the most prevalent molecules throughout the growth process of silkworms. We described AAs classification, occurrence, metabolism, and functions. Online datasets revealed that the essential AAs (EAA) level in fish meal and silkworm pupae (SWP) is comparable. SWP have a high content of methionine and lysine, which are the principal limiting AAs in fish diets, indicating that SWP have nutritional potential to be added to fish diets. Additionally, an overview of the data analyzed displays that SWP have a higher protein efficiency ratio than fish meal, the classical protein-rich source (>1.19 times), and compared to soybean meal, the second-most preferred source of protein in aquaculture (>2.08 times), indicating that SWP can be considered effective for animal feeding. In this study, we provide an overview of the current knowledge concerning AAs, paying special emphasis to EAAs and explaining, to some extent, certain mechanisms and functions of these compounds, from mulberry leaves to larvae–pupae and fish diets.

## 1. Introduction

The silkworm *Bombyx mori* is a fascinating natural phenomenon belonging to the Lepidoptera species, representing the second-most diverse group of insects [1]. For centuries, the silkworm was known for producing silk, a valuable commodity for the textile industry. Improving silk production was for a long time one of the sericulture industry’s main goals. Nevertheless, the production of silk fibers worldwide is decreasing, despite its many benefits as a superior material for clothing, medical, and technological textiles, because of the development of chemical fibers. In the textile business, viscose and polyesters have partially replaced silk [2]. Meanwhile, scientists have been investigating novel applications of the by-products resulting throughout the chain value of sericulture other than silk thread [3]. Silkworms are subjected to successive complex metamorphosis throughout their four life stages (egg, larva, pupa, and adult). After the silk fiber is reeled, a valuable protein-rich by-product known as silkworm pupae (SWP) are generated [4]. SWP represent 60% of the cocoon weight [5] and are considered a complete source of nutrients such as protein (55.6% dry matter (DM)), fats (20–40% DM bases), minerals, vitamins, chitin, as well as an essential source of bioactive compounds [phenols, flavonoids, amino acids (AA), fatty acids (FA)]. Before metamorphosis into SWP, silkworm larvae take from mulberry leaves 72–86% of AAs associated with lipids and FAs, carbohydrates, cellulose, vitamins and minerals, and flavonoids. The positive effects of these bioactive compounds have been demonstrated in the pharmaceutical, healthcare, and animal feeding industries, among other fields [3,6,7,8,9].

Protein (the polymer of AAs) is the main nutrient required by silkworms for growth, development, the production of cocoons and eggs, and the secretion of silk threads [3,4,10,11,12].

Several authors [3,11,13,14,15,16] have reported a considerable variation in the level of protein in SWP (45–95% ± 13.7 as DM bases).

Mahanta et al. [4] state that the proteins in SWP are hydrolyzed and converted into a range of physiologically active compounds, including peptides produced by protease action. The peptides contain AAs involved in several physiological functions [17,18]. Throughout the digestive system, proteins that are consumed by larvae are digested into AAs, which are subsequently required for growth and development [19].

Regardless of all the AAs found in nature, only twenty (α-AA) are used to build protein blocks [20]. The larvae and SWP proteins are characterized by a high biological value, containing 18 types of AA, of which eight are essential (EAA) for human health [9,16,21]. Ten non-EAAs are also suitable for human requirements. SWP have higher phenylalanine and proline contents than hen eggs [16]. Except for isoleucine and leucine, AAs contained by SWP recorded a higher level than soy protein isolate [21].

It has been shown that SWP oils are a great source of polyunsaturated FAs (PUFAs), of which more than 30% are α-linolenic. This has attracted a lot of interest for its potential for medicine applications and the livestock industry [3,7].

SWP are also a rich source of Ca (81 mg), Fe (2.6 mg), K (34.0 mg/g), and Zn (36 μg/g), and contain a low Na/K ratio (0.08), all of which are crucial for human and animal nutrition and health [7,13]. Furthermore, the SWP’s heavy metal concentration is below the limits advised for use in animal feed or for human consumption [7,22].

Although SWP have a high nutritional value, 30–40 years ago these by-products were frequently discarded as waste material or used as fertilizer after the reeling process [5]. Being one of the most valuable sericulture waste products, recycling and reuse solutions were sought. Thus, a part of waste pupae have been used directly for fish feeding or farm animals [23], while in Asian countries they were used mostly for human consumption. The issue is how to redirect this protein waste and valorize it for other industries. As is well known, plants provide a substantial amount of protein for animals, with soybeans being the most popular rich protein source (40–48% in meals).

While there are numerous similarities between fish nutrition and the nutrition of terrestrial animals, there are also major differences in the issues faced by researchers studying fish nutrition. These difficulties are caused mainly by the aquatic medium, which implies paying attention to feed intake and monitoring, waste product collection and quantification, and the distinct physiologies of different fish [24].

In aquaculture, results are also excellent by using plant protein in the diets; however, a few inherent issues have been observed, like the presence of anti-nutritional substances and an inefficient composition of AAs [25]. Given fish meal’s and soybean meal’s availability, productivity, and environmental impact, previous studies have highlighted the need to find alternative sources with elevated protein levels [26]. Thus, insects are new potential protein-rich alternatives which can take the lead in a fish diet with beneficial nutritional and health effects [25]. Considering the fish requirements for protein are 2–4 times higher than in another farm animal and the fact that any protein excess is costly, many studies about fish nutrition are focused on protein intake and metabolism [27,28]. The reasonable price and nutritional qualities are arguments for considering SWP an attractive dietary ingredient for fish. This opportunity provides an innovative perspective for valuing sericulture by-products more effectively. Since mulberry leaves are the unique feed source available to silkworms, they are an essential source of nutrition. There are still gaps in the knowledge regarding flow analysis up to the use in fish, with a focus on AAs, despite publications on the chemical composition or phyto-chemical, pharmacological, and toxicological effects, or on the biochemical variances depending on the types [29,30,31].

This overview covers general knowledge regarding AAs (metabolism, digestion, absorption, roles, physiological processes, and applications) as well as more specifics related to fish and silkworms. In-depth exploring of the passage of protein and its AAs from mulberry leaves through the silkworm larvae–pupae phase to fish, focusing on the findings of their cellular and molecular mechanisms for different functions, was performed. However, a comparative analysis is often difficult, due to the wide range of methodologies and the characteristics of many other fish species. A few pertinent general factors have been considered in this review. Apart from contributing to deepening our knowledge about AAs mechanisms and functions, this paper is an important resource for future studies on the silkworm *B. mori*.

## 2. Materials and Methods

Available online English-language publications were considered regardless of publication date, such as Web of Science, MDPI, PubMed, Research Gate, Google Scholar, and Elsevier database. We have included certain publications written before 2000 because of their contextual relevance. To facilitate the collection of information, the following keywords were used: *silkworm; amino acids; mulberry leaves; pupae; larvae; fish* and a combination of words such as *amino acids metabolism; digestion and absorption of amino acids to silkworm; amino acids requirements of fish; bioactive compounds in silkworm and health; amino acids profile; pupae application to fish*.

The data about the AAs composition of silkworms’ life cycles, starting with mulberry leaves, the larvae’s single source of feed, and of fish, were collected from an online database and statistically descriptively analyzed using IBM SPSS (2011).

A series of limitations and constraints in this study were caused by the lack or incomplete data that would have allowed the use of unitary measurement units, the specific conditions, and the applied methods, which varied between bibliographic sources and species. To emphasize the variability among the sources, the data are displayed as means as well as minimum and maximum values or ranges. Asparagine and glutamine were included in aspartic and glutamic acid when they were provided separately due to their conversion, in the acid-catalyzed esterification stage, into glutamic and aspartic acids, respectively [32].

The protein quality was determined using the equation proposed by Ying [33]: protein efficiency ratio (PER) = (0.06320 × ƩAA) − 0.1539.

## 3. Classification, and Mechanisms of the Amino Acids

AAs are organic natural substances containing C, H, O, and N. Certain AAs, such as methionine and cysteine, contain S. 

There are various criteria used to classify AAs, including chemical structure and biochemical processes such as ketogenesis or glucogenic AAs [34]. Wu, in 2013 [35], proposed a classification of AAs in three groups: basic (arginine, histidine, cysteine), acidic (acid glutamic and aspartic), and neutral.

According to Ito [10], AAs can be classified into five categories: (1) EAAs (group one: arginine, histidine, isoleucine, leucine, lysine, methionine, phenylalanine, threonine, tryptophan, and valine); (2) EAAs (group two: aspartic and glutamic acids); (3) semi-EAAs (proline); (4) non-EAAs (group one: alanine, glycine, serine); (5) non-EAAs (group two: cysteine and tyrosine).

Khan et al. [36] proposed six categories: (1) non-polar AAs (aliphatic), including alanine, valine, leucine, isoleucine, glycine (the simplest structure), and proline (which contains an imino group but not an NH_2_ group); (2) aromatic AAs (phenylalanine, tyrosine, and tryptophan); (3) polar, uncharged AAs, including serine, threonine, cysteine, asparagine, and glutamine; (4) acidic AAs that contain 2 COOH groups: one α-COOH and another β- or γ-COOH group; (5) basic AAs that contain an α-NH_2_ group (histidine, lysine, and arginine); and (6) acid-base AAs, e.g., alanine AA, which are nonpolar, and the -COOH group can be deprotonated.

Ademola [37] has taken into consideration the molecular structure of AAs and classified them into seven groups: (1) AAs with aliphatic side chains (glycine, alanine, valine, leucine, and isoleucine); (2) AAs with -OH group side chains (serine, threonine); (3) AAs with S atom (cysteine and methionine); (4) AAs with side chains containing acidic groups or their amides (aspartic and glutamic); (5) AAs with basic groups (lysine, histidine, arginine); (6) AAs with side chains containing aromatic rings (histidine, phenylalanine, tyrosine, and tryptophan); and finally (7) AAs with imino acids (proline).

In 2021 and then in 2022, Li et al. and Oonincx and Finke [38,39] proposed a more straightforward classification of AAs from a nutritional point of view as either essential or non-essential. The AAs that constitute true protein are grouped under this classification. Taking into consideration that in this review our intention was to describe AAs conversion starting with mulberry leaves, continuing to larvae and pupae up to fish nutrition, the last classification was used.

The following 11 proteinogenic AAs cannot be de novo synthesized by animal cells: cysteine, histidine, isoleucine, leucine, lysine, methionine, phenylalanine, threonine, tryptophan, tyrosine, and valine. As a result, they have been categorized as EAAs nutritionally required for maintenance, growth, development, and survival. On the other hand, the C skeletons of the eight proteinogenic AAs, such as alanine, asparagine, aspartate, glutamate, glutamine, glycine, proline, and serine, can be synthesized de novo by animal cells, and these EAAs are the primary source of nitrogen for these cells [12].

AAs (linked together by peptides) are involved in many physiological processes of the body due to their presence in the chemical composition of hormones, enzymes, and peptides (physiologically active functions), as well as with pharmacodynamic effects by various catabolism products.

Biologically, AAs represent the key substances for protein biosynthesis and low-molecular-weight substances [12,24], participating in the structural edifice of living matter and its energetic and dynamic changes. The protein has a three-dimensional structure determined by its AAs sequence. These structures can be used for various purposes, including immunological defense, mechanical support, mobility, ligand transport, nerve impulse transmission, and the regulation of growth and differentiation.

Alanine, arginine, asparagine, aspartate, cysteine, glutamate, glutamine, glycine, histidine, isoleucine, leucine, lysine, methionine, phenylalanine, proline, serine, threonine, tryptophan, tyrosine, and valine are protein-creating AAs [12]. Non-proteinogenic AAs (e.g., taurine) fulfil essential physiological functions but have yet to be adequately synthesized in many fish species.

High amounts of free AAs perform additional roles in neuronal transmission, detoxification, phospholipid synthesis, energy production, and morphogenetic processes, all of which have significant biological functions and involvement in protein synthesis [19]. According to several studies, AAs have species- and dose-dependent impacts on insect development.

Many investigations on silkworm AAs metabolism have relied on the production of silk. The AAs found in the structure of silk proteins are distinctive: glycine, alanine, serine, and tyrosine make up over 85% of the overall AAs content [40].

The AAs content of SWP complies with FAO/WHO [41] standards established for humans, having appropriate proportions [9,16]. Literature data have mentioned a level of 2.2–6.5% minimum and maximum values, respectively, for methionine and 2.9–9% minimum and maximum values, respectively, for lysine. Mainly, SWP contain a high level of methionine and lysine, which are limiting for monogastric animals, and which suggests the pupae can be beneficial protein alternatives.

## 4. Metabolism of the Amino Acids and Their Functions in Silkworms and Fish

### 4.1. AAs Metabolism in Silkworms

The biology of the silkworm *B. mori* is one of the most advanced among Lepidoptera [42]. In the *B. mori* L. silkworm, the growth rate of organs varies at every stage of development and differentiation. Larvae transition from the immature to the adult stage is based on cellular differentiation mechanisms with the involvement of protein metabolism [43].

The mulberry leaves are a feed source for silkworms. Nutrients from mulberry leaves are ingested and broken down in the silkworms’ midgut, where digestive enzymes are produced. Epithelial cells of the midgut of the silkworm larvae contain columnar, goblet, and stem cells [44]. The goblet cells secrete the digestive fluid, and the cylindrical cells absorb the digested feed. The protein and AAs are used via digestion, absorption, and metabolism processes [38].

Apart from differences observed in Lepidoptera regarding midgut morphological structure (anterior, middle, and posterior regions), differences have been noted in enzyme activity and AAs transport [45]. The digestive juice of the silkworm *B. mori* contains alkaline proteases. Protease activity in the midgut exhibits significant alterations during the transitions from larva to pupa and pupa to adult. Three fractions of these proteases have been identified, isolated, and partially purified. Further, two classes of alkaline proteases have been divided into smaller groups, followed by the AAs sequence [45].

Regarding the proteolysis mechanism in the silkworm digestive system, research has shown that there is a functional difference in the hydrolysis of proteins between the digestive juice and the midgut tissue. In the digestive juice, proteases break down proteins into peptides, and the peptidases act in the midgut tissue by hydrolyzing peptides into AAs [46] (cited by Shinbo et al. [45]).

Shinbo [45] stated that the digestion occurs in the midgut’s anterior region. By using a diet without protein, Shinbo [45] identified endogenous AAs in the midgut (a high concentration of glycine was observed, for example). In the midgut, the AAs absorption rate increases steadily. After being broken down by proteases, proteins are absorbed from the gut lumen as small peptides. Aminopeptidases then liberate AAs in the gut epithelium. The finding shows that peptidase activity is relatively constant across the entire length of the midgut of *B. mori* larvae, and that absorption of the digested small peptides occurs along this whole length.

In 1966, Shyamala and Bhat [47] mentioned that lysine and arginine exhibit a high absorption rate, while a low rate was recorded for aspartic acid and, most notably, glutamic acid. Leucine, valine, and methionine are slowly absorbed by silkworm larvae. Compared to all cumulative AAs, glycine’s absorption ratio changes very distinctly. It remains high throughout the entire midgut, including the anterior region [46].

The intracellular AAs play an essential role in protein synthesis associated with their role in phospholipids synthesis, as a source of energy, or support of morphogenetic processes (transformation of specific larval tissues in adult tissue by cell differentiation) that occur throughout the silkworm’s life cycle [43,48]. If the insect does not receive any EAAs, its ability to synthesize proteins may be affected.

The general silkworm metabolic pathways are the same as in other animals: (i) transamination process; (ii) interconversion between certain AAs. The first information about AAs transamination reaction in the muscle, fat body, gut, and Malpighian tubules of the silkworm *B. mori* was given by Bheemeswar and Sreenivasaya [49] and Fukuda [50], as cited by Chen and Bachmann-Diem [51].

Bheemeswar [52] identified in the silkworm larvae an enzyme system capable of converting aspartate to alpha-alanine, which is important for the precursor synthesis of silk fibroin. The enzyme activity in the Malpighian tubules is higher than in the fat body, but it appears to be lower in the larvae midgut. In 1964, Chen and Bachmann-Diem [51] highlighted that the insect hemolymph contains free AAs and related compounds. Later, in 2021, Murugesh et al. [48] revealed that the total hemolymph, a reservoir of nutrients, energy, and metabolic intermediates [40], is 100–300 times higher than that of human blood.

Moreover, serine, glycine, and alanine interconversion have been demonstrated by Ito [10]. These AAs have a vital role in the synthesis of silkworm tissue and in cocoon silk production as well. The threonine dehydrogenase converts threonine to glycine [40]. In mammals, pyruvate is recognized as an important metabolic intermediary between proteins and carbohydrates. Fukuda [50], cited by Bricteux et al. [53], demonstrated the pyruvate transamination leading to alanine synthesis in the silk gland of *B. mori*.

It appears that none of the EAAs from group one [10] eliminated from an artificial diet are produced in sufficient levels to support larval growth and development. These AAs are generated in small amounts from other AAs or specific precursors. Thus, some studies have shown that citrulline is the precursor of arginine but in insignificant quantity. Although the silkworm may convert phenylpyruvate to phenylalanine and indole lactic acid to tryptophan, it is also highly improbable that these precursors will be present in the silkworm in significant quantities [10].

Ito [10] showed that larval growth was somewhat impacted when aspartic or glutamic acids were omitted from the diet; however, when they were eliminated, the larval growth was significantly suppressed. In the larvae tissues, glutamic and aspartic acids are interconverted by transaminase. Aspartic and glutamic acids are involved in glycine and alanine synthesis (the main AAs of silk protein) [10].

The larvae grow slowly without proline acid, and larvae in the fifth instar, fed on a diet lacking in proline, also produce a smaller cocoon. In silkworms, proline synthesis can be achieved from its precursors through the ornithine cycle. Glutamic acid, arginine, or ornithine contributed to covering the need for proline; however, an excess of arginine in place of proline indicated a detrimental effect. Since proline is classified as a semi-EAA, proline is likely synthesized at an insufficient level [10].

The alanine, glycine, and serine AAs were included by Ito [10] in non-essential group one, having less effect on larval development, proved by the dietary elimination of any of them. Each of them added to a diet deficient in glutamic and aspartic acids and increased dietary efficiency to a certain extent, but not as much as when acidic AAs were added. Moreover, adding all three to a diet rich in acidic AAs slightly enhanced the growth of larvae and synthesis of silk [10]; however, some research has indicated that glycine plays a critical role in controlling the silk synthesis [40], revealing that AAs have significant regulatory functions for silk fiber secretion. Glycine may decrease urea levels in the hemolymph and increase the efficiency with which AAs are converted into silk protein [40]. According to Tang et al. [54], the BmGT1-L transporter gene in the sericin gland was identified in the midgut microvilli and continuously expressed throughout the feeding phases. Adequate amounts of glycine associated with ectopic expression of the BmGT1-L gene in the posterior silk gland increase silk yields in silkworm-fed mulberry leaves. Glycine acts via two mechanisms: (1) enhances endomitotic DNA synthesis in silk gland cells via PI3K/Akt/TOR signaling; (2) stimulates silk synthesis as a raw material.

It was determined that cysteine and tyrosine (non-EAAs group two) were not required, their absence little affecting larval growth. The nutritional importance of these AAs cannot be neglected given their correlation with the dietary levels of phenylalanine and methionine [10]. The dietary addition of methionine and phenylalanine can be metabolized by the larvae to cysteine and tyrosine.

The metabolism of glucose, tryptophan, and organic acids is affected by the AAs.

According to Laufer [55], cited by Mahmoud Souad et al. [56], many, if not all, of the hemolymph proteins are enzymes. The fat body provides most of the AAs required for silk production through the hemolymph, which is essential for transportation and maintaining the intricate balance of free AAs during metabolic processes. The disruption of AAs metabolism in the larva hemolymph raises urea and uric acid levels and increases the amounts of crude proteins in the pupae while the efficiency of protein conversion into secretory silk proteins decreases [40].

### 4.2. Amino Acids Metabolism in Fish

Fish tissues are mostly made up of proteins, which account for 65–75% of the total on a DM basis [57].

According to Wu et al. [20], cited by Li et al. [38], five processes are involved in the synthesis of proteins in fish and other animals: (1) gene transcription; (2) translation; (3) peptide elongation; (4) termination; (5) posttranslational changes [35].

Generally, fish species have a simple digestive system. But, while some fish still have short, relatively basic tracts, others have longer, more complex ones. Even as adults, several fish species do not have stomachs. The fish that lack a stomach belong to the microphagous class [58]. The tract is separated into three sections: the midgut, the distal or hindgut, which ends in the rectum and the foregut [58]. The mucosa, submucosa, and muscularis are the three main sections that make up the gut. The gastrointestinal tract becomes increasingly complex during the larval and juvenile stages.

Feed intake, digestion, and absorption of the digested substances are fundamental in converting available nutritive substances from feed to energy and organic matter for fish survival [59,60]. However, energy resulting from a surplus of protein is considered a vital component for all body cells. Furthermore, the primary factor influencing fish weight gain appears to be protein deposition.

Like silkworms, enzymes play an important role in digestion and must be appropriate and in adequate quantities. In 1995, Jürss and Bastrop [61] highlighted that different enzymes have important functions in the breakdown and transamination of AAs, which facilitates the regulation of the different uses of specific kinds of AAs. Digestive fluid enzymes and gut epithelial cells break down proteins, lipids, carbohydrates, and nucleic acids into smaller molecules that are easier to absorb and assimilate.

Firstly, the proteins are broken down in the stomach (except fish without a stomach). Enzymes become active once they are secreted into a pH-regulated environment that is favorable to digestion functions. There have been no conclusive findings that fish stomach juice contains lipase and amylase.

Pepsin, brush-border peptidases, and pancreas-derived peptidases are involved in the various phases of protein digestion. When these enzymes act together, protein can be almost entirely decomposed (digestibility varies from 70% to 97%) into individual AAs, dipeptides, and tripeptides [62]. Pepsins hydrolyze peptides, with specificity for phenylalanine and tyrosine. The secretion of pepsinogen depends on dietary protein levels [24].

Borlongan et al. [58] specified that fish digestion depends on the structure of the intestines’ absorptive cells. Transamination may compensate for non-EAAs imbalances in dietary protein and might also be crucial for enhancing protein utilization [63].

The intestinal cells primarily produce their digesting enzymes, including amylase and maltase, aminopeptidases, esterase, and alkaline and acid nucleosidases, on the brush edge of the epithelium.

Protease enzymes hydrolyze proteins into specific AAs or polypeptide chains. This process can occur either in acidic or basic pH [58]. Pancreatic and intestine proteases can more easily digest proteins thanks to the endopeptidase activity of gastric juice. Trypsin and chymotrypsin play a vital role in the digestion of proteins in the intestine. Carboxypeptidases further divide the polypeptides that result from their interaction. In the gut lumen, the chymotrypsin and trypsin act synergically to produce small peptides and protein fragments, which are then further hydrolyzed by pancreatic and brush-border exopeptidases. Thus, low molecular weight peptides and AAs are produced in the gut lumen during protein breakdown. Cytoplasmic peptidases further break down very small peptides in free AAs. However, it is unclear if these small peptides also enter the systemic circulation.

Protease, lipolytic, and amylase dietary composition have been demonstrated to differ between fish species. While lipolytic activity does not respond to changes in proteolytic and amylase activity for certain species, amylase and/or protease activity appears to be unresponsive for other species [24]. Upon complete digestion, the midgut is the primary site of absorption for several nutrients.

The development of the defense system against pathogens depends on the absorption of intact proteins, which appears to occur most frequently in the distal intestine [24].

Certain AAs function as precursors for other compounds. The precursor of cysteine is methionine. Methionine also provides CH_3_ groups for numerous different compounds, including choline and creatine. Tyrosine is created when an OH group is added to phenylalanine. In the urea cycle, arginine is converted to ornithine. Histamine is created when a COOH group is taken off from histidine. The precursor of serotonin is tryptophan [64].

Intestinal absorption of AAs is complex and requires a multitude of transporters on the brush-border membrane of the enterocyte that are largely specialized for various AAs or classes of AAs. Enterocyte development, as well as the concentrations of particular AAs in the intestine, impact the efficiency of the absorption process [65].

Several endogenous and dietary proteins escape proteolytic digestion in the proximal portions of the colon. These proteins could be taken up as macromolecules.

## 5. Amino Acids Composition

### 5.1. Amino Acids Profile throughout the Chain Value of Silkworm B. mori L.

If considering the environmental strategic assessment based on the estimation of CO_2_ sequestration in mulberry leaves, silkworm rearing (larvae fed by mulberry leaves) for fish diet is economically considered not viable; however, in our opinion we must take into consideration that silkworm larvae have a brief growing period as well as the mulberry leaves’ rapid recovery. Thus, the mulberry’s significance for sequestering carbon is slightly affected. Furthermore, mulberries have long been utilized as feed for farm animals and as a source of nutrients for silkworm larvae to obtain silk.

Although this tree’s ecological significance was neglected over time, its relevance for sustainable development has gained attention. This plant has grown in popularity recently because it is known to be a medicinal plant, a multipurpose plant for environmental safety, and an industrial plant employed in many different industries [66]. In our opinion, some mulberry tree types can be used as an alternative to certain classical feedstuff to feed animals (fish as well) since they require fewer resources such as regular irrigation and substantial applications of fertilizer, beyond its use in sericulture, and in other industries to generate income and create jobs.

Data screening from many authors’ studies allow us, by using statistical analyses, to compare the AAs composition of mulberry leaves, larvae, and pupae (Table 1). Thus, in Table 1 we present the AAs composition (mean ± standard deviation, SD, minimum and maximum values) starting with mulberry leaves and continuing to larvae and pupae.

The wide range of values observed and the large variability of the online datasets are worth mentioning. Our data show that pupae protein value is 12.5% higher than in larvae. While glutamic acid recorded higher values for pupae and mulberry leaves, unexpected glycine was the most predominant AA on larvae. A possible explanation consists of the complexity of factors that influence the AAs profile, such as the treatment and methods of analyses, varieties of mulberry leaves and/or silkworm breed/hybrid, sampling, etc. It is well known that limited AAs for fish are lysine, methionine, and threonine. In the pupae, e.g., lysine represents about 6.3% of the total sum, methionine represents 4.14%, and threonine represents 5.12%. The PER of pupae is greater than that of the mulberry leaves or larvae (2.54 times higher than in larvae and 3.56 times higher than in mulberry leaves). This value allows us to speculate that pupae may transform into an alternative source of nutrition for other species.

Significant qualitative and quantitative differences in biochemical characteristics linked to proteins, phenolics, and minerals were found when genetically European mulberry cultivars were compared [31]. Using multivariate analysis, Krajnc et al. [31] defined distinct chemotypes and evaluated them according to the composition of single AAs and phenolics. The information would be helpful in selecting and growing the best native European cultivars with a high concentration of vital AAs and a particular phenolic component. In practical terms, information gathered from many sources can be used to direct one or other types for purposes such as raising silkworms or even therapeutic purposes. At the same time, according to Selih et al. [78], total proteins, total phenolics, derivatives of caffeoylquinic acid, and flavonols are all significantly impacted by regional dispersion. The utilization of linear discriminant analysis facilitated an all-encompassing evaluation of leaf metabolites and validated the unique biochemical characteristics of mulberries originating from various regions of Slovenia. Caffeoylquinic acid, isoquercetin, rutin, kaempferol acetylhexoside, and quercetin malonylhexoside were identified as important feed markers based on their shown positive effects on the growth and quality of cocoons in silkworm larvae. Irrespective of the variety of mulberry leaves, all had a sufficient amount of nutrients for the silkworms’ bodies to function normally [29]. Through an examination of the current genetic resources for mulberries and their leaf metabolites, Selih et al. [78] emphasized the significance of the white mulberry for science, culture, and the environment. The active biocompounds content of the mulberry leaves is further found in the pupae. Thus, silkworm pupae have been shown to contain a total of eighteen distinct phenolic acid compounds, mostly ferulic acid and cinnamic acid, and fifteen distinct flavonoid components, such as luteolin, quercetin, myricetin, epicatechin, and catechin. A thorough examination of the nutritional and bioactive components in silkworm pupae produced a large number of chemicals that could be investigated for use in feed additives, functional foods, medicines, and nutraceuticals [79].

### 5.2. Amino Acids Composition in Fish

Table 2 shows the AAs composition in fish.

The EAAs have a concentration <14.8% to that of non-EAAs. The most predominant EAAs are leucine and lysine. The lysine level is around 1.93 times lower than SWP, whereas methionine records a level 3.02 times lower than SWP.

Methionine is the first limiting AA, followed by lysine [86]. The requirements vary between species. Methionine and lysine levels must be elevated for commercial feed when other protein sources, particularly plant proteins, are substituted for fish meal. Dietary lysine is crucial for growth rates and immune and gastrointestinal system development [87].

Fish had a more favorable AAs composition than milk, beef, or eggs [83]. The most predominant AA in fish’s whole body is glutamic acid (7.21%); however, the minimum value determined was 0.2 g (Table 2).

According to Mohanty et al. [82], glutamic acid is involved in transamination reactions and is required for the synthesis of glutathione, which is needed for the elimination of highly toxic peroxides and polyglutamate folate cofactors as well.

### 5.3. Factors Influencing Availability and/or Absorption of AAs

To our knowledge, no research has investigated the availability and transfer of AAs from mulberry leaves to silkworm larvae and pupae to fish. The leaf position, season, and variety can impact protein and AAs digestibility during the third through fifth silkworm larvae instars [88]. Proteins from insects are often high digestible. According to Longvah et al. [89], cited by Wu et al. [7], the AAs score and protein digestibility-corrected AAs score of silkworm pupae are 100 and 86, respectively. Because chitin contributes to the crude protein, only 73% of the overall protein content is truly digestible. Nevertheless, chitin makes up just 3–4% of DM [90].

Fish diets have reported great success when fed plant protein; nonetheless, a number of inherent issues have been found, such as the presence of anti-nutritional factors and an unbalanced AAs composition [25].

There are some risks (AAs imbalance or deficit) in attempts to totally replace fish meal with other protein sources in fish diets, so some cautions are necessary. A more accurate understanding of the biological value is provided by the true availability values of AAs, feed composition being more accurately known and cost-effective.

According to Xing et al. [91], at least three factors must be considered in order to diversify the protein sources required for the large number of aquatic species: (i) the profile, bioavailability, and sources of AAs; (ii) the amount and balance of AAs; (iii) the ideal ratios of AAs in diet to digestible energy. Since excess AAs contribute to energy production, we need to understand more about the relative roles of individual AAs oxidation and its favored use in relation to total metabolic demands.

A crucial inquiry concerns the absence of a stomach to first-feeding fish larvae and how this impacts their ability to digest feed. From a practical standpoint, this raises questions about the limitations on the absorption of AAs and their consequences for first-diet composition. The stomach’s main function in adult fish is to partially digest chyme and break down nutritive substances that can be released into the midgut under controlled conditions [92,93]. However, since pancreatic and intestinal enzymes facilitate enzymatic protein digestion, the absence of a stomach does not impair this process in fish larvae.

Unbalances between the dietary and fish larval body protein AAs profiles may be linked to an AAs loss of more than 40% of the total amount of AAs in the diet, since body proteins constitute the only source of AAs storage in larvae [94]. One of the varying factors that can improve protein digestibility in the fish gastrointestinal system is treatment applied during the feed processing.

When represented as a percentage of dietary protein, there is a substantial positive association between the level of dietary EAAs incorporated and the required amount of EAAs [91].

## 6. Amino Acids Requirements

### 6.1. Requirements of Silkworm Larvae

The silkworm larvae are the only stage at which feed is required. To enhance both larval growth and cocoon yield, it is crucial to know the AAs requirements and the ratios between EAAs and non-EAAs. The AAs metabolic functions are connected to nutritional requirements. Furthermore, silkworm nutrition is important in many aspects as it is related to an organism’s physiological functions and health. Supporting nutritional requirements is one way to ensure the vital nutrients ingested for normal metabolism, development, and, ultimately, health.

It is well known that mulberry leaves provide significant amounts of vital nutrients, particularly protein and AAs [95]. According to Raghuvanshi et al. [96], proteins, carbohydrates, vitamins, sterols, and minerals are the primary nutrients found in mulberry leaves. The crude protein composition is generally similar to that of most legumes.

A crude protein level ranging from 15 to 28% was determined by many authors [96,97,98,99,100,101,102]. As noted by Srivastava et al. [103], beyond the high concentration of crude protein (15.31–30.91%), mulberry leaves contain fat from 2.09 to 4.93%, carbohydrates from 9.70 to 29.64%, energy from 113 to 224 kcal/100 g, ash from 14.59 to 17.24%, and neutral detergent fiber from 27.60 to 36.66%.

It is worth mentioning that mulberry leaves provide 72–86% of larvae AAs requirements [104]. There is a positive correlation between daily requirement and mulberry leaves composition. The optimum amount of protein in the diet for growth is 22–26%, which is comparable to mulberry leaves [105]. Furthermore, mulberry leaves also provide the amount of each of the ten EAAs (arginine, histidine, isoleucine, leucine, lysine, methionine, phenylalanine, threonine, tryptophan, and valine) that silkworm larvae require to survive. Over 60% of the AAs ingested by silkworm larvae are used in silk production.

Notable for their rich nutrients and functional chemical content, mulberry leaves are gaining recognition for their various health benefits. The issue relies on the reduction of the mulberry plantation area. One of the consequences is that artificial diets have been proposed as suitable alternatives. Even though the artificial diet has gained greater acceptance in recent years, studies have revealed that the development of silkworms has been affected [106]. It has been shown that mulberry leaves have a higher nutritional content than any artificial diet [40].

Studies on the feed intake are typically limited to the fourth and fifth instars of silkworm larvae since, during these stages, 80% of the total leaves are consumed [107]. Machii et al. [68] highlighted that silkworm larvae require for growth a minimum of 8 mg/g DM, methionine 4 mg/g DM, threonine 7 mg/g DM, valine 8 mg/g DM, isoleucine, leucine, arginine, and phenylalanine 8 mg/g DM each, and histidine 5 mg/g DM.

Horie [105] mentioned the silkworm larvae ingest approximately 56.9 mg protein/g body weight/day (utilized 67%), lysine 4.3 mg (used 81.4%), methionine 0.7 mg (utilized 85.7%), and threonine 3.8 mg (utilized 78.9%). The rate of absorption is high.

Nutrient conversion efficiency in silkworms depends upon environmental factors, feed quality, and feed quantity. Feed conversion efficiency is regarded as a crucial physiological parameter for assessing the superiority of silkworm breeds and directly or indirectly contributes to an important aspect of the cost–benefit ratio of feeding silkworms [107]. The growth, development, feed consumption, utilization, and conversion efficiency of different breeds of silkworms differ.

An adequate quantity of AAs is requested for enzyme structures and acting as transport receptors [105].

Murugesh et al. [48] used the AAs serine, glycine, and alanine to estimate the minimum concentration of specific AAs involved in larvae and cocoon characteristics. Providing to silkworm larvae (1–5 instar up to spinning) glycine, alanine, and serine supplements (10, 100, and 100 ppm concentration) enhanced the larval growth and characteristics of the cocoon.

Dietary conditions, such as the absence or alteration of a particular AA, influence the pattern of hemolymph AAs and the rate of AAs’ conversion. Metabolic adaptation is required for this kind of dietary regulation. Combining active compounds like enzymes, hormones, and other compounds may regulate and control the substance for the silkworms’ metabolic and physiological functions.

Adult silkworm females need protein for ovaries and eggs and proper secretion of juvenile hormones. The adult silkworm males typically do not need protein for sperm. The dietary needs vary with age, gender, and physiological stress [107].

One possible cause of reduced conversion efficiency in formula feed is an unbalanced AAs composition. Nevertheless, it is still unknown how the molecular makeup of AAs in feed affects silk output [40].

### 6.2. Requirements of Fish

Fish species differ from other living organisms more than any other phylum in terms of the anatomy and histomorphology of the digestive system [24,108,109]. Certain fish species utilize their feed more efficiently than terrestrial animals; however, this is not always the case [38].

As for the silkworm, EAAs for fish must be supplied through diet, while non-EAAs can be synthesized de novo from α-keto acids or through transamination or from other AAs [63].

Different digestive enzymes break down dietary proteins, which can be converted into free AAs. The digestive tract absorbs the released free AAs, which are then utilized by different tissues to synthesize tissue proteins. The quality of the protein determines the requirements. A “*balanced*” AAs composition leads to a lower demand. Since the amount of protein consumed and the requirement for AAs are always correlated, the requirement might alternatively be stated as a percentage of total protein in the diet. There is a variation in fish species’ capacities to use free AAs. Since crystalline AAs are employed to alter dietary levels, this could make it challenging to measure requirements.

Conceição et al. [63] mentioned that notwithstanding recent advancements, little is known about the protein and AAs needs of fish larvae. There is evidence that diet composition influences the growth, development, and productivity of aquatic animals [38]. To formulate effective diets, the distinctive traits of fish and physiological processes must be considered.

Colin et al. [110] mentioned that the starting point of the first comprehensive research on fish’s nutritional requirements was marked in the 1950s. Although fish do not really need a true protein, they require a balanced diet that includes both EAAs and non-EAAs.

Fish EAAs requirements for maintenance, protein retention, catabolism, and fecal losses, were frequently determined by classical dose–response feeding methods. These approaches were performed to quantify protein and EAAs requirements based on the analysis of dose–response curves by using weight gain as a response criterion [24].

After that, the minimal dietary requirement was determined to be the lowest level of EAAs that maximizes live weight gain. Along with weight increase, protein and EAAs depositions are also being used as response parameters. The state of the art is still somewhat superficial and dispersed for different fish species [24,111]. The applicability to real-life conditions has been questioned due to significant limitations in the experimental design, poor diet characterization, and poor growth performance achieved in most studies [111].

The factorial approach was a valuable tool for determining fish requirements, which started to gain attention as one of the primary methods for predicting AAs requirements. This approach considers an alternative diet and experimental design to get a graded intake of AAs and/or protein to calculate the necessary amounts for maximal protein gain or growth as well as for maintenance. This one includes the need at the ration level, is applicable in specific conditions, and is based on accurate data on AA absorption efficiency, maintenance AA requirements, whole-body AA accretion rates, and efficient AA utilization beyond maintenance [24,112]. The impediment was given by the high degree of variability attributed to several factors: differences between and within species, design, and conditions of the experiment or to the variety of mathematical and statistical techniques used in the estimation of requirements.

Based on an integrated growth and nutrient utilization model to estimate EAAs and considering the effects of biological and dietary factors on AAs consumption and demand, Hua and Bureau [111] developed a new factorial AAs requirement model for salmonid fish. This model estimates the EAAs requirement for economically feasible feeds more precisely.

From a qualitative viewpoint, the radioisotope approach developed by Cowey et al., referenced by Millamena [64], consisted of fish injected intraperitoneally with radioactively labelled ^14^C glucose and fed a natural diet for 7 days. After that, the fish protein was separated. After hydrolyzing a sample of the separated protein, the individual AA was separated using chromatography and their radioactivity was quantified.

The average value of EAAs needs for fish (juveniles and on-growing fish) are shown in Table 3. The average protein required for fish ranged between 30.0–62.0%, with a mean value of 42%, close to that mentioned by Teles et al. [27], while the dietary level of crude protein varies between 24–55% [24,27].

For example, lysine is the most predominant AA required for fish (1.8% of total EAAs). According to Wang et al. [86], fish have a methionine demand that varies depending on species, life stage, methionine forms, and amounts of cystine and taurine in the diet. It ranges from 0.49% to 2.5% of the diet (1.49% to 4.7% of dietary protein).

Animal feeding has gained more and more importance in crystalline AAs. DL-methionine, made by chemical synthesis; L-lysine, produced by fermentation; L-threonine and L-tryptophan, produced in the 1980s; and L-isoleucine and L-valine, made in the 1990s, have started to be used on a large scale.

As stated in FAO Annex 4 [114], arginine, histidine, isoleucine, leucine, lysine, methionine, phenylalanine, threonine, tryptophan, and valine are required for fish. However, the AAs contents of fish species vary little [115]. Nearly 90% of AAs detected in the ileum are absorbed. Tryptophan and glycine record lower digestibility.

The main distinction is a reduced ability to absorb and/or digest complex proteins and AAs requirements.

According to Li et al. [116], growing fish recorded lower protein retention rates than growing pigs and chickens. One possible explanation could be that fish species require more protein, contributing the most energy required for metabolic processes. AAs, as substrates for energy metabolism rather than carbohydrates, are highly dependent on dietary protein to meet fish metabolic needs [117]. In fact, the ideal dietary nutritional protein levels in fish are greater than those in terrestrial agricultural animals.

The relationship between protein level and N excretion has also raised concerns [38,118]. Following the start of feeding, the digestive system plays a crucial role in providing AAs required for the rapid development of fish larval tissues. Entire protein digestion is only available after several weeks. Fish larvae require feed with a higher molecular N and a higher protein digestibility [63]. The amount of N in each AA varies. As a result, the protein levels provided in feed compositional tables are never totally accurate.

Since protein and AAs are the primary components of tissue growth, they are also the most expensive nutrients used in animal production. For this reason, the development of feed for aquatic organisms depends significantly on protein.

Fish meal is the primary protein source in aquaculture, with high protein digestibility and AAs bioavailability. Nonetheless, 90% of the fish used to make fish meal might be directed to human food [29]. Due to the high cost of fish meal, less expensive protein sources have been used to meet the fish needs. Many alternative protein sources used for fish feeding do not possess the EAAs profile that fish meals provide [22].

## 7. Silkworm Pupae in Fish Feeding

While formulating diets, greater attention must be paid to ensuring EAAs requirements to fish. Meanwhile, for protein quality, the PER was used in this study by considering the requirement of EAAs.

Since fish meal has a balanced AAs content, good palatability, and high digestibility, it is a primary feed ingredient for fish. Furthermore, fish meal has a rich supply of vitamins, minerals, and long-chain omega-3 FAs necessary for healthy animal growth [119]. However, the availability of fish meal has rapidly decreased due to overfishing, the constant fall in wild fish captures, and the quick expansion of aquaculture [87,118]. New feed ingredients are required for aquaculture due to a lack of fish meal, leading to higher fish feed costs [25].

Table 4 presents mean and range values (as measures of variability) of the composition of three protein-rich sources for fish feeding (fish meal, a classical source; soybean meal, the second-most preferred source of protein in aquaculture; and SWP as a potential alternative). Data collected from online databases on the AAs composition of the three ingredients show a higher concentration of EAAs in SWP (>1.15 times compared to fish meal Σ EAA and >1.83 times that of soybean meal). Furthermore, the PER is higher in SWP than fish meal (>1.19 times) and compared to soybean meal (>2.08 times).

Many authors have used various protein-rich plants to replace total or partial fish meals, like rapeseed meals, soybean meals, palm meals, and peanut meals, which are affordable [118,133]. For example, in 2024, Yang et al. [118] assessed how various dietary protein sources (a mixture of soybean meal and rapeseed meal as the plant-derived protein, and a mixture of fish meal and soybean meal with rapeseed meal as the mixed protein) affect the intestinal absorption, antioxidant capability, growth performance, and muscle quality of triploid Crucian carp. The findings offered a theoretical base for the sensible use of plant-derived protein in aquatic feed, which is necessary for the development of aquaculture in a sustainable manner. Still, the results showed a better impact of a diet based on fish meal as a source of animal protein.

In the feed industry, soybean meal was regarded as the “*gold standard*” intact protein source [128]. Given that methionine and lysine are often the AAs that limit the growth of fish, soybean meal’s superior AAs profile enhances cereal grains’ value in the formulation of a diet. The versatility and availability of prices were the issues raised in using soybean meal. For example, Romania depends on the unpredictable import of soybean meal at a variable cost. Drought frequency may also cause a more considerable discrepancy between feed availability and animal nutrients [3].

Since conventional feeds account for 70% of livestock production costs, insect-based animal diets have attracted interest [134]. Insects are a desirable alternative for animal feed due to their high nutritional content, short life cycles, high productivity, high feed conversion factors, little spatial requirements, and minimum environmental impact [133]. In July 2017, the European Union passed Regulation (EU) 2017/893, an amendment to Regulation (EC) No 999/2001 [135], which permits the use of aquafeed-processed animal protein resulting from 7 insects. The European Food Safety Authority (EFSA) (2015) released a scientific opinion on a risk profile concerning the production and use of insects for food and feed [136]. After the silk has been reeling, pupae should be processed and transformed into animal protein for utilization in diets for animal farms. Following the addition of the silkworm *B. mori* to the list of insect species permitted for use in animal feed, its utilization was stated to be suitable. As a result, Annex X to Regulation (EU) No 142/2011 [137] was changed appropriately.

Thus, many animal diets started to include insects [3,134,138]. SWP is a promising feed ingredient. Several studies have highlighted the multiple implications of SWP for health [3,16,20,89] and the impact on human and animal feeding [16,76,90,139].

The protein and AAs profile of SWP is superior to that of soybean meal and comparable to that of fish meal, as the literature data collected reveals (Table 4). For instance, methionine levels in SWP are 38% greater than in fish meal and 81% higher than in soybean meal. Lysine content is 45% higher than in soybean meal and 4.2% lower compared to fish meal. The average protein content is comparable to that of fish meal.

Research using de-oiled SWP meal showed that it is a better source of protein for fish diets and has minimal impact on the performance and growth of farmed fish species [25,140].

Begum et al. [141] used SWP in varying proportions mixed with clam meat to replace 25, 50, 75, and 100% of the protein from fish meal. Dietary SWP associated with clam meat enhanced fish growth compared to fish meal. The greatest rise in the PER occurred when 50% of the fish meal was substituted with SWP x clam meat. In the opinion of Bergum [141], the association between rich-protein feedstuffs can be more effective. Later, in 2019, it was highlighted that mixing insect meals with a complementary source with a valuable nutritional profile, or adjusting the substrate utilized as a nutrient source, can increase FA content, digestibility, and even palatability, and it is possible to increase the nutritional value of insects. Hăbeanu et al. [3] mentioned that SWP is additionally valuable due to an excellent profile in FAs, especially PUFA, particularly from the n-3 group. It is known that a significant number of datasets emphasized the importance of PUFA and essential FAs for the health of humans and animals. Given their superior essential FAs composition, associated with a valuable profile in AAs, it became evident that SWP might be employed in diets for both humans and animals.

Shakoori et al. [142] studied the impact of replacing fish meal with varying levels of SWP (5%, 10%, and 15% SWP) on rainbow trout’s hematological parameters over 60 days. The results show that SWP stimulates the immune system of rainbow trout by increasing the number of white blood cells. Nevertheless, intake of SWP might result in a drop in red blood cell and hemoglobin levels, which can lead to anemia symptoms.

Zhou et al. [140] investigated the implications of feeding a fermented meal combination of wheat, rapeseed, and SWP (FMM, *wt:wt:wt*, *1:1:1*) at various levels (0, 40, 80, 119, and 160 g × kg^−1^) to mirror carp for 58 days to replace fish meal protein. The issue raised consists of unwanted odor caused by a high concentration of readily peroxidized lipids (80–100 g × kg^−1^ free FAs and over 700 g × kg^−1^ unsaturated FAs) in SWP which determines a decrease in the animals’ sensory quality, via SWP mixed with rapeseed and wheat. In brief, the growth, feed intake, feed protein efficiency ratio, serum superoxide dismutase and malondialdehyde, and the relative expression of TNF-α and IL-6 in the hepatopancreas were not impacted by replacing fish meal with FMM at a dose of 40 g × kg^−1^. Conversely, higher protein replacement of fish meal with FMM, such as 80, 120, and 160 g × kg^−1^, resulted in decreased growth, higher serum glutamic oxaloacetic transaminase and the relative expression of TNF-α and IL-6 in the hepatopancreas.

According to Raja [25], without compromising growth or quality, SWP are a good feed component for trout, catfish, Indian major and exotic carp, and common carp.

## 8. Conclusions

The relevance of AAs and their impact on the sericulture value chain up to fish feeding were the primary concerns of this study, since AAs are essential molecules for every living organism and the most critical compounds throughout silkworm rearing phases.

Although we acknowledged in our previous research the special significance of FAs on the sericulture chain, protein and AAs also have a major impact, both functionally and quantitatively. Beyond other nutrients, EAAs are present in amounts that enable us to acknowledge the various valences of sericulture products. This starts with the AAs profile of mulberry leaves, continues with their uptake by larvae through the pupal stage, and ends with their valorization in fish diets. The greater concentrations of protein and AAs found in SWP are comparable to or even better than those found among different possible raw materials for aquaculture. The presence of lysine, methionine, and threonine in SWP limits the utilization of synthetic AAs in fish diets, reducing feed cost, and provides one more argument for the potential implications of products derived from sericulture for other sectors.

There are still many challenges (availability, price, industrial pollution) and unanswered questions in our knowledge, especially concerning the digestibility of AAs unique to each species and breed, the value of AAs in larval stages for species mono- or polivoltine, the impact of seasons on the composition of AAs in mulberry leaves, etc.

Even though the artificial diet used for silkworm larvae has gained approval lately, more research is needed to identify low-cost industrial waste that can be used to supplement mulberry leaves with AAs in a way that is appropriate for all species, including silkworm larvae, from an economic and environmental perspective.

## Figures and Tables

**Table 1:** Composition of amino acids in mulberry leaves and silkworm *B. mori* larvae and pupae.

Amino Acids % DM *	Value ± SD	Mulberry Leaves	Larvae	Pupae
Protein	Mean	23.55 ± 0.35	52.87 ± 6.83	60.47 ± 2.36
	Minimum	11.75	22.59	26.0
	Maximum	37.36	68.0	95.0
**EAAs**				
Lysine	Mean	2.09 ± 0.30	2.45 ± 0.14	6.01 ± 0.64
	Minimum	1.0	2.14	1.37
	Maximum	5.0	2.93	9.09
Methionine	Mean	0.54 ± 0.12	0.96 ± 0.16	3.94 ± 0.48
	Minimum	0.21	0.64	0.45
	Maximum	1.89	1.42	6.50
Threonine	Mean	1.54 ± 0.25	2.45 ± 0.33	4.88 ± 0.54
	Minimum	0.77	1.97	1.14
	Maximum	4.0	3.77	9.70
Arginine	Mean	1.27 ± 0.07	2.48 ± 0.33	4.22 ± 0.54
	Minimum	0.88	1.95	1.06
	Maximum	5.0	3.80	6.80
Histidine	Mean	0.89 ± 0.21	1.72 ± 0.48	6.63 ± 1.73
	Minimum	0.35	1.08	0.66
	Maximum	3.56	3.65	25.0
Valine	Mean	1.83 ± 0.32	2.82 ± 0.43	4.67 ± 0.37
	Minimum	0.85	2.29	1.29
	Maximum	5.0	4.55	6.18
Phenylalanine	Mean	1.80 ± 0.30	1.82 ± 0.08	4.14 ± 0.53
	Minimum	0.91	1.73	0.88
	Maximum	5.0	2.08	7.33
Isoleucine	Mean	1.54 ± 0.29	1.52 ± 0.24	3.49 ± 0.39
	Minimum	0.74	1.18	0.83
	Maximum	5.0	2.25	5.70
Leucine	Mean	2.45 ± 0.34	2.52 ± 0.20	5.55 ± 0.69
	Minimum	1.45	1.98	1.20
	Maximum	7.0	3.06	8.30
Tryptophan	Mean	0.36 ± 0.02	0.40 ± 0.03	1.22 ± 0.21
	Minimum	0.26	0.33	0.34
	Maximum	0.54	0.44	1.76
**Σ EAAs**		**14.31**	**19.14**	**44.75**
**PER**		**0.75**	**1.05**	**2.67**
**Non-EAAs**				
Aspartic acid	Mean	2.57 ± 0.27	4.56 ± 0.14	7.91 ± 1.13
	Minimum	1.50	4.15	1.50
	Maximum	5.0	4.84	11.69
Glutamic acid	Mean	3.23 ± 0.59	4.50 ± 0.32	14.39 ± 1.10
	Minimum	1.0	3.82	5.69
	Maximum	11.00	5.10	21.50
Alanine	Mean	1.46 ± 0.04	6.10 ± 1.54	5.52 ± 0.49
	Minimum	1.09	2.59	2.45
	Maximum	1.58	9.78	10.20
Glycine	Mean	2.0 ± 0.4	7.11 ± 2.20	5.58 ± 0.56
	Minimum	0.86	2.21	1.89
	Maximum	6.0	12.08	10.09
Proline	Mean	0.75 ± 0.10	1.52 ± 0.21	5.20 ± 0.63
	Minimum	0.33	1.14	2.04
	Maximum	1.31	2.08	9.76
Serine	Mean	1.42 ± 0.25	4.60 ± 1.10	5.04 ± 0.59
	Minimum	0.66	2.25	2.22
	Maximum	4.0	6.76	10.6
Tyrosine	Mean	0.80 ± 0.02	3.39 ± 0.51	5.61 ± 0.47
	Minimum	0.62	2.50	2.24
	Maximum	0.89	4.60	8.46
Cysteine	Mean	0.20 ± 0.02	0.54 ± 0.07	1.13 ± 0.15
	Minimum	0.11	0.43	0.20
	Maximum	0.30	0.76	1.90
**Σ non-EAAs**		**12.43**	**32.32**	**50.38**

* EAAS, essential amino acids; PER = protein efficiency ratio calculated with the equation from Ying et al. [33]: PER = (0.06320 × ƩAA) − 0.1539. Sources: Machii and Katagiri [67]; Machii et al. [68]; Yao et al. [69]; Wang et al. [70]; Al-Kirshi et al. [71]; Astuti et al. [72]; Olteanu et al. [73]; Rao [8]; Zhou and Han [13]; Tassoni et al. [32]; Anootthato et al. [74]; Kwon et al. [75]; Akande et al. [76]; Tomotake et al. [21]; Ji et al. [77]; Ying et al. [33].

**Table 2:** Composition of protein and amino acids in fish.

Items *	Mean ± SD	Minimum	Maximum
Proteins **%**	19.20 ± 1.09	16.0	31.0
***EAA, g/100 g protein***			
Lysine	3.54 ± 0.71	0.09	16.10
Methionine	1.38 ± 0.23	0.02	4.0
Threonine	3.10 ± 0.50	0.30	7.90
Arginine	1.55 ± 0.27	0.10	6.50
Histidine	2.31 ± 0.39	0.03	7.90
Valine	3.27 ± 0.54	0.05	8.60
Phenylalanine	2.51 ± 0.41	0.06	6.30
Isoleucine	2.69 ± 0.43	0.20	6.50
Leucine	4.44 ± 0.69	0.40	10.40
Tryptophan	1.48 ± 0.40	0.10	6.50
**Σ EAAs**	**26.27**		
***Non-EAAs***			
Aspartic acid	6.83 ± 0.86	0.10	12.30
Glutamic acid	7.21 ± 1.12	0.20	16.55
Alanine	3.75 ± 0.53	0.08	8.10
Glycine	6.21 ± 1.39	0.10	32.0
Proline	1.37 ± 0.37	0.07	9.60
Serine	3.83 ± 0.52	0.09	7.20
Tyrosine	1.48 ± 0.42	0.20	8.40
Cysteine	0.16 ± 0.03	0.04	0.40
**Σ non-EAAs**	**30.84**		

* Essential amino acids (EAA); Sources: Bechtel [80]; Bechtel et al. [81]; Mohanty et al. [82]; Cieslik et al. [83]; Elavarasan [84]; Fatma et al. [85].

**Table 3:** Essential amino acids requirements of fish.

Items	Mean ± SD	Minimum	Maximum
Proteins, **%**	42.10 ± 1.20	24.0	62.0
EAA, % protein *			
Lysine	5.18 ± 0.16	3.7	6.23
Methionine **	3.04 ± 0.20	2.0	4.0
Threonine	3.22 ± 0.35	0	4.50
Arginine	4.66 ± 0.18	3.30	6.0
Histidine	1.88 ± 0.06	1.50	2.20
Valine	3.43 ± 0.09	2.80	4.0
Phenylalanine	5.16 ± 0.51	3.40	6.50
Isoleucine	2.91 ± 0.18	2.20	4.40
Leucine	4.20 ± 0.22	3.30	5.30
Tryptophan	0.68 ± 0.06	0.30	1.4
**Σ EAAs**	**34.67**		

* Essential amino acids (EAA); ** Only references where methionine was evaluated separately from cysteine and phenylalanine without tyrosine were considered. Sources: Wilson and Halver [113]; Millamena [64]; Mohanty et al. [82]; Teles et al. [27]; Radhakrishnan et al. [28].

**Table 4:** Mean ± SD of protein and amino acids composition in fish meal, soybean meal, and pupae (g/100 g protein, range value).

Specification *		Fish Meal	Soybean Meal	Pupae (SWP)
Protein	Mean	64.45 ± 2.27	47.05 ± 0.76	60.47 ± 2.36
	*Range*	20.0	5.50	69.0
*EAA*				
Lysine	Mean	6.28 ± 0.55	3.25 ± 0.18	6.01 ± 0.64
	*Range*	4.33	4.06	7.72
Methionine	Mean	2.44 ± 0.19	0.73 ± 0.06	3.94 ± 0.48
	*Range*	1.34	1.06	6.05
Threonine	Mean	3.64 ± 0.28	1.95 ± 0.13	4.88 ± 0.54
	*Range*	2.37	2.54	8.56
Arginine	Mean	5.15 ± 0.39	3.98 ± 0.29	4.22 ± 0.54
	*Range*	3.51	4.30	5.74
Histidine	Mean	2.43 ± 0.22	1.51 ± 0.11	6.63 ± 1.73
	*Range*	1.99	1.81	24.34
Valine	Mean	4.25 ± 0.33	2.55 ± 0.15	4.67 ± 0.37
	*Range*	2.43	2.76	4.89
Phenylalanine	Mean	3.44 ± 0.26	2.78 ± 0.22	4.14 ± 0.53
	*Range*	2.06	3.08	6.45
Isoleucine	Mean	3.68 ± 0.28	2.49 ± 0.17	3.49 ± 0.39
	*Range*	2.14	2.63	4,87
Leucine	Mean	6.30 ± 0.47	4.29 ± 0.32	5.55 ± 0.69
	*Range*	3.62	4.90	7.10
Tryptophan	Mean	0.93 ± 0.10	0.73 ± 0.08	1.22 ± 0.21
	*Range*	0.48	0.91	1.42
**Σ EAAs**		**38.54**	**24.26**	**44.75**
**PER**		2.28	1.38	2.67
***Non-EAA***				
Aspartic acid	Mean	7.95 ± 0.67	6.0 ± 1.30	7.91 ± 1.13
	*Range*	3.70	9.38	10.19
Glutamic acid	Mean	11.30 ± 0.97	10.09 ± 1.78	14.39 ± 1.10
	*Range*	5.64	11.21	15.81
Alanine	Mean	5.66 ± 0.38	2.38 ± 0.37	5.52 ± 0.49
	*Range*	2.16	2.76	7.77
Glycine	Mean	6.62 ± 0.82	2.38 ± 0.42	5.58 ± 0.56
	*Range*	5.50	2.69	8.20
Proline	Mean	4.07 ± 0.43	2.74 ± 0.49	5.20 ± 0.63
	*Range*	2.75	3.13	7.72
Serine	Mean	3.48 ± 0.30	2.64 ± 0.55	5.04 ± 0.59
	*Range*	1.85	3.47	8.38
Tyrosine	Mean	2.62 ± 0.27	2.0 ±0.29	5.61 ± 0.47
	*Range*	2.09	2.49	6.22
Cysteine	Mean	0.67 ± 0.08	0.64 ± 0.09	1.13 ± 0.15
	*Range*	0.70	1.46	1.70
**Σ non-EAAs**		**42.37**	**28.87**	**50.38**

* Essential amino acids (EAA); PER = protein efficiency ratio calculated with the equation from Ying et al. [33]: PER = (0.06320 × ƩAA) − 0.1539. Sources: Jørgensen et al. [119]; Donadelli et al. [120]; Gamboa-Delgado et al. [121]; Cho and Kim [122]; Ido and Kaneta [123]; Zheng et al. [124]; Barone et al. [125]; Wang et al. [126]; Thakur et al. [127]; Dozier and Hess [128]; Lagos and Stein [129]; Karr-Lilienthal et al. [130]; Haghbayan and Mehrgan [131]; Elavarasan [84]; Raja et al. [25]; Rahimnejad et al. [132].

## Data Availability

The data presented in this study are available on request from the corresponding authors.
